# Porcine Alveolar Macrophages’ Nitric Oxide Synthase-Mediated Generation of Nitric Oxide Exerts Important Defensive Effects against *Glaesserella parasuis* Infection

**DOI:** 10.3390/pathogens8040234

**Published:** 2019-11-13

**Authors:** Qi Cao, Huan Wang, Wenbin Wei, Yujin Lv, Zhao Wen, Xiaojuan Xu, Xuwang Cai, Huanchun Chen, Xiangru Wang

**Affiliations:** 1 State Key Laboratory of Agricultural Microbiology, College of Veterinary Medicine, Huazhong Agricultural University, Wuhan 430070, Hubei, China; cq20100804@163.com (Q.C.); pinkapplewh@163.com (H.W.); wenbWei@163.com (W.W.); 80775@hnuahe.edu.cn (Y.L.); witerswenzhao@163.com (Z.W.); xuxiaojuan@mail.hzau.edu.cn (X.X.); caixuwang@mail.hzau.edu.cn (X.C.); chenhch@mail.hzau.edu.cn (H.C.); 2 Key Laboratory of Preventive Veterinary Medicine in Hubei Province, The Cooperative Innovation Center for Sustainable Pig Production, Wuhan 430070, Hubei, China; 3 Key Laboratory of Development of Veterinary Diagnostic Products, Ministry of Agriculture of the People’s Republic of China, Wuhan 430070, Hubei, China; 4 International Research Center for Animal Disease, Ministry of Science and Technology of the People’s Republic of China, Wuhan 430070, Hubei, China; 5 College of Veterinary Medicine, Henan University of Animal Husbandry and Economy, Zhengzhou 471000, Henan, China

**Keywords:** *Glaesserella parasuis*, porcine alveolar macrophages, nitric oxide, NOS2, NF-κB signaling

## Abstract

*Glaesserella parasuis* is a habitual bacterium of pigs’ upper respiratory tracts. Its infection initiates with the invasion and colonization of the lower respiratory tracts of pigs, and develops as the bacteria survive host pulmonary defenses and clearance by alveolar macrophages. Alveolar macrophage-derived nitric oxide (NO) is recognized as an important mediator that exerts antimicrobial activity as well as immunomodulatory effects. In this study, we investigated the effects and the signaling pathway of NO generation in porcine alveolar macrophages 3D4/21 during *G. parasuis* infection. We demonstrated a time and dose-dependent generation of NO in 3D4/21 cells by *G. parasuis*, and showed that NO production required bacterial viability and nitric oxide synthase 2 upregulation, which was largely contributed by *G. parasuis*-induced nuclear factor-κB signaling’s activation. Moreover, the porcine alveolar macrophage-derived NO exhibited prominent bacteriostatic effects against *G. parasuis* and positive host immunomodulation effects by inducing the production of cytokines and chemokines during infection. *G. parasuis* in turn, selectively upregulated several nitrate reductase genes to better survive this NO stress, revealing a battle of wits during the bacteria–host interactions. To our knowledge, this is the first direct demonstration of NO production and its anti-infection effects in alveolar macrophages with *G. parasuis* infection.

## 1. Introduction

*Glaesserella parasuis* is the causative agent of porcine Glässer’s disease, which is characterized by polyarthritis, fibrinous polyserositis and meningitis [1,2]. It is considered one of the most important opportunistic bacterial pathogens in nursery piglets, with the ability to cause high morbidity and mortality in China [3]. It is an early colonizer of the upper respiratory tract and part of the normal microbiata of healthy pigs [4]. Under certain circumstances, some highly virulent strains can invade the lungs and the circulatory system, and cause the subsequent multiple-systemic polyserositis [5].

Based on the infection dynamics, *G. parasuis* infections initiate from the invasion and colonization of the lower respiratory tract of pigs, and breakthrough host pulmonary defenses and clearance [6]. During these interaction processes, *G. parasuis* has to compete with lung-resident alveolar macrophages, which play essential roles in the first-line of host defense. This mainly involves the production and release of pro-inflammatory factors, such as interleukin-8 and macrophage inflammatory protein-1β; and antimicrobial bioactive molecules, such as reactive oxygen species or reactive nitrogen species (RNS) [7,8,9]. Usually, the innate immune system employs pathogen-associated molecular patterns (PAMPs), such as Toll-like receptors and nucleotide oligomerization domain-like receptors, to detect bacterial products and trigger innate immune responses [10,11].

Nitric oxide (NO) production is an important mechanism of the mammalian innate immune response [12]. Generally, mammalian cell NO is production from L-arginine catalyzed by three nitric oxide synthase (NOS) isoforms: neuronal NOS (NOS1), endothelial NOS (NOS3) and inducible NOS (NOS2) [13,14]. NOS1 and NOS3, mainly expressed in neurons and endothelial cells, respectively, catalyze the low generation of NO that is specifically involved in the regulation of neuronal cell differentiation or microvascular permeability [15,16]. In contrast, NOS2 is widely distributed in multiple cell types, and is significantly induced under certain infection or inflammatory stimulations via PAMPs [17,18]. For example, microbe-induced NOS2 production can be facilitated by myeloid differentiation factor 88 and the caspase adaptor recruitment domain family member-9-mediated nuclear factor (NF)-κB signaling pathway in a calcium-independent manner [19,20].

The antimicrobial activity of NO and NOS2 has been reported within macrophages and other myeloid cells in many studies [21,22]. NO, catalyzed by NOS2, reacts with structural elements, components of replication machinery, nucleic acids, metabolic enzymes and virulence-associated molecules of infectious pathogens [21]. It inactivates the enzymatic activity of the Fe–S metalloproteins, and mediates NO-dependent killing of *Burkholderia mallei* [23]. NO also interferes with the tricarboxylic acid cycle to inactivate the dihydrolipoyl dehydrogenase component of α-ketoglutarate dehydrogenase in *Salmonella enterica* serovar Typhimurium [24]. Moreover, NO treatment combined with amoxicillin and clavulanic acid enhanced the ex vivo killing of *Streptococcus pneumoniae* in adenoid tissue [25]. However, elevated levels of NO from the persistent activation of NOS2 may lead to adverse effects on the host; for example, allograft rejection, septic shock and neurodegeneration [26,27,28]. Additionally, the NO produced by NOS2 catalyzation plays an important role in the development of osteoarthritis, in which NO overgeneration inhibits matrix synthesis and promotes cartilage breakdown and pain [29]. However, little is known about NO generation in alveolar macrophages in response to *G. parasuis* infection. The specific effects of NO involvement in antimicrobial activity and host innate immunity against *G. parasuis* have not been investigated.

Here, we report *G. parasuis* infection-induced NO generation in the porcine alveolar macrophage cell line 3D4/21. We investigated both the potential influence and signaling transduction pathway of NO generation in 3D4/21 cells in response to *G. parasuis*. We demonstrated positive host immunomodulation effects by releasing notable NO production in 3D4/21 cells after *G. parasuis* infection. NO showed both inhibitory effects on bacterial growth and immune activation effects on 3D4/21 cells, and *G. parasuis* in turn, selectively altered its gene expression to better survive these detrimental influences. The characterization of NO production and its potential effects in response to *G. parasuis* infection expanded our knowledge of *G. parasuis* pathogenesis from the perspective of pathogens and host interactions, which will better facilitate the prevention and control of this disease.

## 2. Results

### 2.1. G. parasuis SH0165 Infection of 3D4/21 Cells Induces the Production of NO That Depends on Bacterial Viability

**Figure 1:**
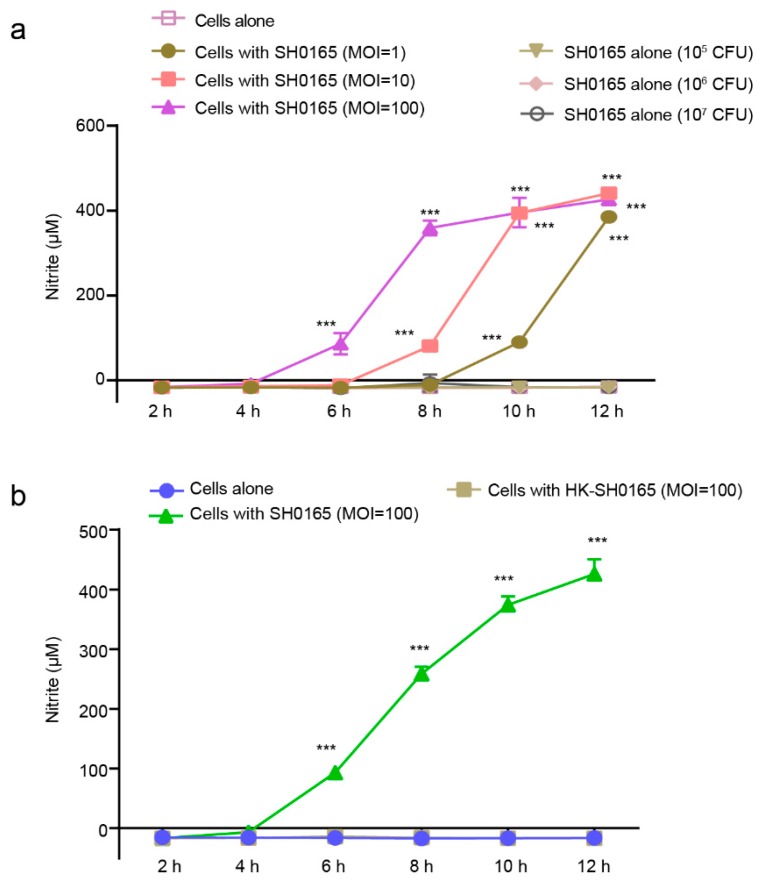
*Glaesserella parasuis* induced the time-dependent and multiplicity of infection (MOI)-dependent production of nitrite in 3D4/21 cells which required bacterial viability. (**a**) 3D4/21 cells were challenged with or without different doses (MOI of 1, 10 and 100) of SH0165 for 12 h. (**b**) 3D4/21 cells were infected with SH0165 (MOI 100) or HK-SH0165 at the same MOIs for 12 h. The supernatants were collected for nitrite measurement at 2 h intervals via the Griess reaction. Cells alone and bacteria alone at different colony forming units were used as controls. Data are shown as the means ± SDs from three replicates. Statistical differences were analyzed using the Student’s *t*-test (***, *p* < 0.001).

The production of RNS by macrophages is recognized as an important part of the host immune defense against bacterial pathogens [30,31]. Here, the porcine alveolar macrophage cell line 3D4/21 was used to investigate possible NO production during its exposure to *G. parasuis* SH0165. NO production was measured and reflected by the accumulation of nitrite in the culture medium via the Griess reaction [32]. As shown in Figure 1a, there was no detectable NO production by 3D4/21 cells or bacteria alone during 12 h of observation. When challenged by the SH0165 strain, a significant induction of NO was observed over time. Interestingly, a faster generation of NO detected following a higher multiplicity of infection (MOI) applied. 

Because only a few prokaryotic organisms have mammalian NOS homologs, such as *Staphylococcus aureus* [33], *Bacillus anthracis* [34,35] and the plant pathogen *Streptomyces turgidiscabies* [36], and the fact that genome sequencing analysis revealed that *G. parasuis* SH0165 (accession number NC_011852.1) lacks bacterial NOS [37], we investigated NO production in culture medium next, by incubating 3D4/21 cells with either viable SH0165 or heat-killed SH0165 (HK-SH0165). We observed a significant and time-dependent induction of NO in 3D4/21 cells in response to viable SH0165 over 12 h of incubation, but found heat-inactivated bacteria were not able to stimulate NO generation (Figure 1b), suggesting its induction strictly depends on the viable SH0165 interaction with host cells. Together, these data suggest that SH0165 strains have the ability to induce the generation of NO in porcine alveolar macrophages, which largely requires bacterial viability.

### 2.2. Cellular Inducible NOS2 and Cationic Amino Acid Transporter 2 (CAT2) are Responsible for NO Generation in 3D4/21 Cells Upon G. parasuis Infection

**Figure 2:**
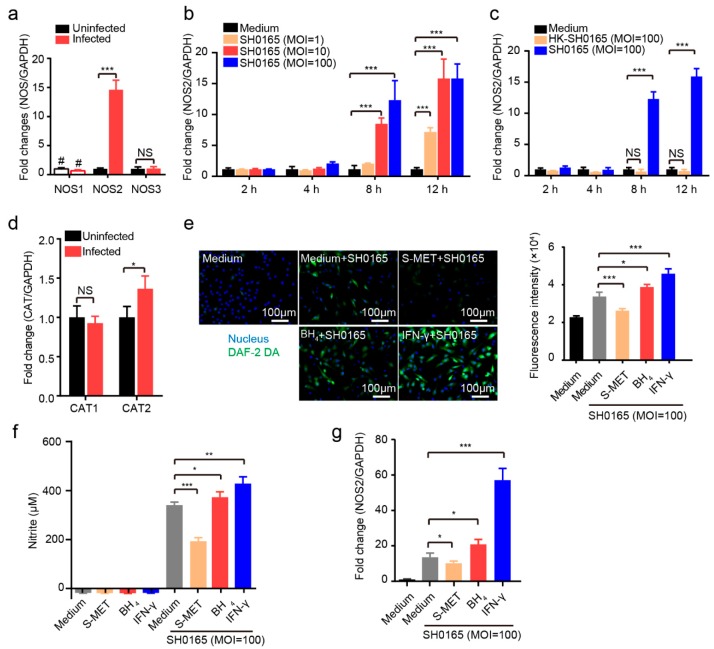
NOS2 and CAT2 facilitated *G. parasuis*-induced NO production. (**a**) Real-time PCR analyzing the expression levels of NOS isoforms in 3D4/21 cells in response to SH0165 infection for 12 h. (**b**) Real-time PCR analysis of NOS2 expression in 3D4/21 cells in response to different infection doses of SH0165 at indicated time points. (**c**) NOS2 expression in 3D4/21 cells challenged with SH0165 or HK-SH0165 at indicated time points were analyzed by real-time PCR. (**d**) CAT expression levels in 3D4/21 cells infected with or without SH0165 for 12 h. (**e**) Intracellular NO generation at 12 h of SH0165 infection at MOI of 100 was detected by the DAF-2 DA probe via fluorescence microscope capture and quantified with a fluorescence microplate reader. Scale bar, 100 μm. (**f**) Nitrite levels from culture supernatants of cells with or without SH0165 infection and other treatments at 12 h via the Griess reaction. (**g**) Real-time PCR analysis and comparison of NOS2 expression in SH0165-infected 3D4/21 cells receiving other treatments. All real-time PCR results were normalized to GAPDH and shown as means ± SDs from three independent assays. Statistical differences were calculated using two-way ANOVA (***, *p* < 0.001; **, *p* < 0.01; *, *p* < 0.05; NS: not significant). #, undetectable.

NO is normally generated via catalyzation of NOS, so we determined which NOS isoform is involved in *G. parasuis*-induced NO production. As shown in Figure 2a, we observed significantly increased expression of NOS2, but not NOS1 or NOS3, in response to 12 h of SH0165 infection. This induction was both time-dependent and bacterial MOI-dependent in 3D4/21 cells in response to SH0165 infection (Figure 2b). We also noticed that the NOS2 induction required bacterial viability because HK-SH0165 was not able to mediate NOS2 upregulation, while viable bacteria significantly induced NOS2 upregulation (Figure 2c). 

Arginine can be transported into the cytoplasm to support NO generation, which requires cationic amino acid transporters CAT1 and CAT2 in macrophages [38,39]. We observed a significant increase in CAT2 in 3D4/21 cells 12 h post infection, while CAT1 was unaffected by the challenge, indicating that cellular CAT2 participates in NO synthesis in response to *G. parasuis* infection (Figure 2d).

To further validate the participation of NOS2 in *G. parasuis*-induced NO production, we applied the NOS2 inhibitor S-MET (50 μM), the NOS2 enzymatic activity co-factor BH4 (50 μM) and the positive stimulator IFN-γ (50 ng·mL^−1^). The membrane-permeable NO probe DAF-2 DA [40] was used to indicate intracellular NO production in 3D4/21 cells upon challenge. As shown in Figure 2e, *G. parasuis* SH0165 infection of 3D4/21 cells significantly increased the generation of intracellular NO, which was prevented by S-MET pre-treatment. Moreover, BH4 significantly enhanced NO synthesis in SH0165-infected cells, while IFN-γ significantly stimulated NO production upon SH0165 challenge. The nitrite concentration was discovered to be, in the culture medium, like that seen following intracellular NO generation (Figure 2f). The analysis of NOS2 transcriptional levels revealed a significant induction of transcription in response to SH0165, which was significantly decreased by S-MET treatment. As expected, both BH4 and IFN-γ positively upregulated NOS2 transcription, which exhibited a similar pattern to that of intracellular NO generation and nitrite production (Figure 2g). Taken together, these findings suggest that *G. parasuis* infection of 3D4/21 cells significantly upregulates NOS2 as well as CAT2 for the induction of NO generation.

### 2.3. NO Exerts an Inhibitory Effect on G. parasuis Growth and Proinflammatory Activation in 3D4/21 Cells

**Figure 3:**
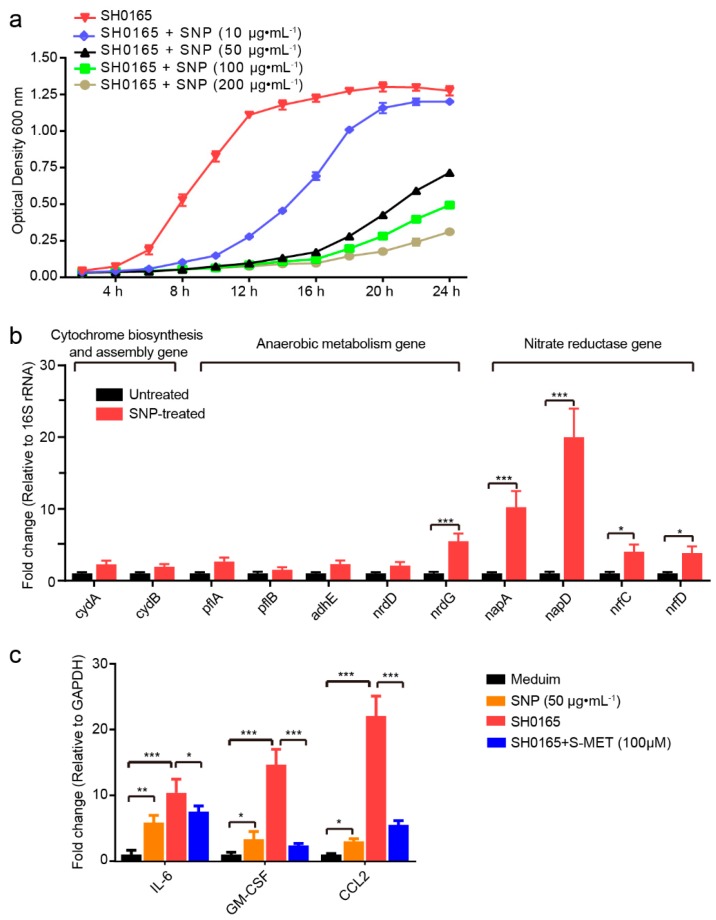
NO reduced the in vitro growth of *G. parasuis* and induced proinflammatory cytokine and chemokine expression in 3D4/21 cells. (**a**) The NO donor SNP exhibited a dose-dependent inhibition on the growth of *G. parasuis* SH0165. (**b**) Real-time PCR detection of multiple NO response-associated genes in *G. parasuis* SH0165, including cytochrome biosynthesis and assembly genes (*cydAB*), anaerobic metabolism genes (*pflAB*, *adhE* and *nrdDG*) and nitrate reductase genes (*napAD* and *nrfCD*). (**c**) Real-time PCR analysis of cytokine and chemokine expression in 3D4/21 cells receiving SNP (50 μg·mL^−1^), SH0165 or SH0165 with pretreatment of S-MET (100 μM) for 24 h. Cells with medium alone were used as the control group. Results were generated from three duplicate assays and were normalized to bacterial 16S rRNA or GAPDH of cells. Statistical differences were analyzed using two-way ANOVA (***, *p* < 0.001; **, *p* < 0.01; *, *p* < 0.05).

Next, we investigated the potential effects of NO on bacteria and cells. Following addition of the exogenous NO donor SNP (10 μg·mL^−1^ to 200 μg·mL^−1^), the exogenous addition of NO significantly inhibited the growth of *G. parasuis* SH0165 in a dose-dependent manner compared with the SH0165 strain alone over a 24 h period (Figure 3a). Since NO showed an inhibitory effect on the viability of *G. parasuis*, we subsequently investigated the potential reactions of *G. parasuis* in response to the NO challenge. The possible metabolic or protein translation-associated genes, and the potential NO-responsive genes, such as the cytochrome biosynthesis and assembly genes, anaerobic metabolism genes, nitrate reductase genes, iron-sulfur cluster repair genes, NO detoxification genes and tricarboxylic acid cycle-involved genes [24,25,41], were tested by using real-time PCR on bacteria at the mid-logarithmic phase receiving 5 μg·mL^−1^ of SNP for 2 h. Our findings showed that SNP treatment significantly upregulated transcription of the anaerobic metabolism *nrdG* gene, and the nitrate reductase *napA*, *napD*, *nrfC* and *nrfD* genes (Figure 3b), revealing a protective feedback of gene regulation in *G. parasuis* when challenged by NO. These data together revealed that NO exhibits a strong bacteriostatic effect and induces a protective adaption of *G. parasuis* by feedback upregulating several NO-responsive genes.

As an important immune mediator, we examined the potential effects of NO on regulating inflammatory cytokines and chemokines in 3D4/21 cells. Cells were treated with or without 50 μg·mL^−1^ SNP or challenged by SH0165 (MOI 100) with or without 100 μM S-MET. The exogenous addition of NO induced significant upregulations of several proinflammatory factors, such as interleukin-6 (IL-6), granulocyte–macrophage colony-stimulating factor (GM-CSF) and chemokine (C-C motif) ligand 2 (CCL2) in 3D4/21 cells, suggesting an elevated inflammatory response mediated by NO. We also observed a significant induction of IL-6, GM-CSF and CCL2 by SH0165 infection in 3D4/21 cells, even higher than that induced by SNP treatment; however, this was largely inhibited by S-MET (Figure 3c), implying that NOS2-mediated NO production contributes to proinflammatory activation in alveolar macrophages during *G. parasuis* infection.

### 2.4. G. parasuis’ Activation of NF-κB Signaling Mediates NO Generation in 3D4/21 Cells

**Figure 4:**
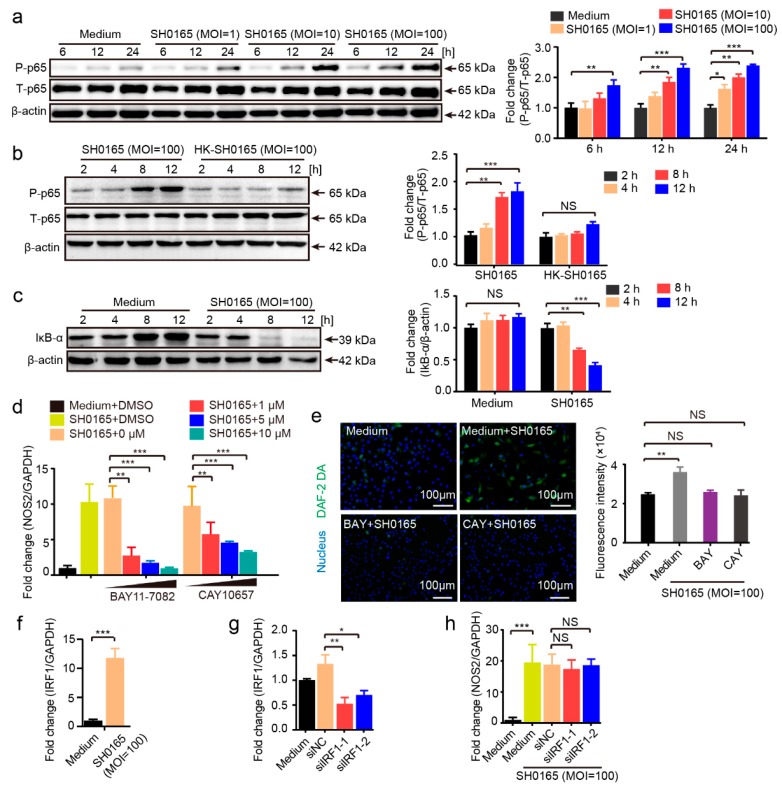
SH0165-induced activation of NF-κB signaling pathway-mediated NO generation in 3D4/21 cells. (**a**) Western blot showing time-dependent and bacterial MOI-dependent phosphorylation of the NF-κB p65 subunit in 3D4/21 cells in response to SH0165. (**b**) Western blot and comparison of p65 phosphorylation in response to SH0165 and HK-SH0165 (MOI 100) at the time points indicated. (**c**) Western blot analysis of IκB-α degradation in whole-cell lysates of 3D4/21 cells with or without SH0165 infection. (**d**) Real-time PCR analysis of NOS2 expression in 3D4/21 cells upon 12 h of SH0165 infection (MOI 100) with or without pretreatment of BAY11-7082 or CAY10657. (**e**) Intracellular NO generation at 12 h of infection at MOI of 100 was detected by the DAF-2 DA probe via fluorescence microscope and quantified with a fluorescence microplate reader. Scale bar, 100 μm. (**f**) Real-time PCR analysis of IRF1 expression in 3D4/21 cells upon SH0165 infection. (**g**) Cells were collected 24 h after siRNA transfection and real-time PCR analysis of IRF1 expression. NC, negative control. (**h**) IRF1 silencing by siRNA and was then infected with SH0165 or non-infected for 12 h. Real-time PCR analysis of IRF1 expression in 3D4/21 cells. Band densitometry was normalized to T-p65 or β-actin and shown as mean ± SD from three independent analyses. All real-time PCR data were normalized to GAPDH and shown as means ± SDs from three independent assays. Statistical differences were analyzed using Student’s *t*-tests (***, *p* < 0.001; **, *p* < 0.01; *, *p* < 0.05; NS: not significant).

Previous studies have reported the activation of NF-κB signaling in PK-15 cells upon *G. parasuis* infection [42]. In 3D4/21 cells, we also observed the significant activation of NF-κB signaling in response to *G. parasuis*, by the demonstration of time-dependent and MOI-dependent phosphorylation of the p65 subunit (Figure 4a). We found that activation of the p65 subunit required bacterial viability because p65 phosphorylation was significantly attenuated when cells were challenged by HK-SH0165 (Figure 4b). We also observed the significant and time-dependent degradation of IκB-α in 3D4/21 cells upon SH0165 infection (Figure 4c), further supporting the fact that *G. parasuis* infection of 3D4/21 cells induces the activation of NF-κB signaling. Two NF-κB signaling inhibitors, BAY11-7082 and CAY10657, significantly decreased SH0165-induced upregulation of NOS2 in a dose-dependent manner (Figure 4d), and significantly attenuated the SH0165-induced intracellular NO fluorescence intensity (Figure 4e).

Interferon regulatory factor 1 (IRF1) overexpression in HEK293T cells was previously shown to enhance the transcriptional activity of endogenous human NOS2 gene expression [43]. To determine whether IRF1 functions in response to *G. parasuis* infection, 3D4/21 cells were incubated with SH0165 (MOI 100) for 12 h, and the expression of IRF1 was evaluated by real-time PCR. As shown, SH0165 infection of 3D4/21 cells significantly upregulated IRF1 (Figure 4f). However, silencing IRF1 expression by siRNA (Figure 4g) revealed that IRF1 knock-down did not significantly decrease NOS2 expression compared with the significant induction by SH0165 infection (Figure 4h). These data, therefore, suggest that the *G. parasuis*-induced increase of NOS2 transcription in 3D4/21 cells largely depends on the activation of NF-κB signaling, and does not occur via IRF1-mediated signaling.

**Figure 5:**
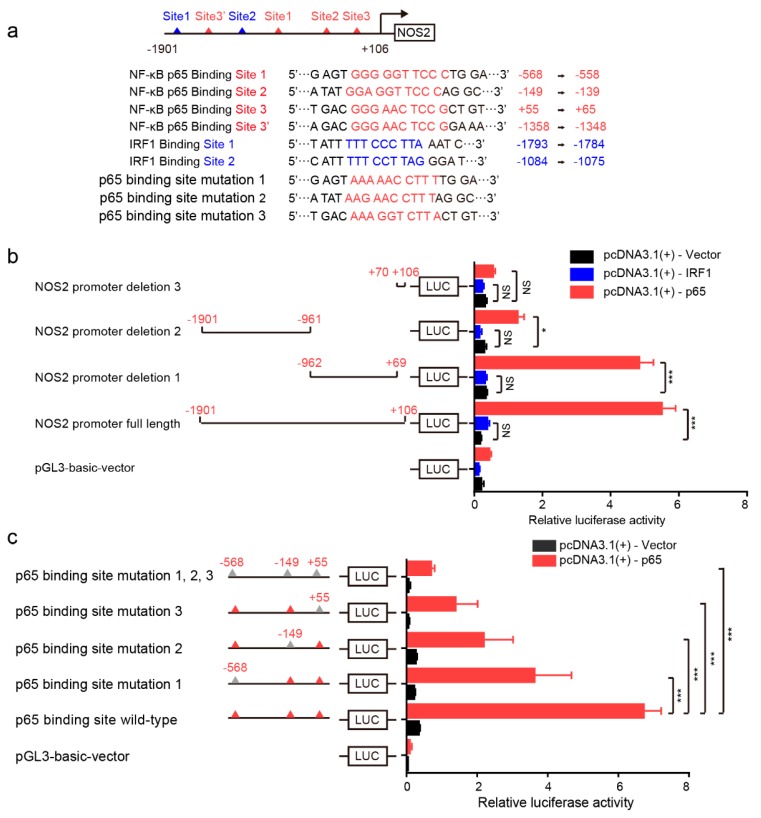
The effects of NF-κB p65 binding sites on the transcriptional activity of the NOS2 promoter via luciferase reporter assays. (**a**) The potential p65 binding sites (red triangles) and IRF1 binding sites (blue triangles) in the NOS2 promoter region. (**b**) The NOS2 full-length promoter region and different truncations were co-transfected with p65 or IRF1 to analyze and compare luciferase activities. (**c**) A set of p65 binding site mutations was introduced into the NOS2 promoter region and transcriptional activities were analyzed by luciferase reporter assays with p65 co-transfection. Firefly luciferase activity was measured and normalized to Renilla luciferase activity. Results are expressed as means ± SDs from six well duplicates. The statistical differences were calculated using two-way ANOVA (***, *p* < 0.001; *, *p* < 0.05; NS: not significant).

To further support the role of NF-κB signaling in mediating NO production, the dual luciferase reporter assay was used to demonstrate the direct regulation of NF-κB signaling in NOS2 expression. As shown in Figure 5a, four potential NF-κB p65 binding sites (red triangles) and two potential IRF1 binding sites (blue triangles) on the promoter region of NOS2 were bioinformatically predicted (Figure 5a). Of these, NF-κB p65 Binding Site 3 appeared twice, at +55 to +65 and −1358 to −1348 (Figure 5a). Based on the location of these binding sites, we constructed a luciferase reporter plasmid containing the full-length NOS2 promoter region (~1.9 kb) and three different mutations. Deletion 1 ranged from −962 to +69 and contained three potential NF-κB p65 binding sites (sites 1, 2 and 3); deletion 2 ranged from −1901 to −961 and contained two potential IRF1 binding sites and one potential NF-κB p65 binding site (Site 3); and deletion 3 ranged from +76 to +106 and contained no NF-κB p65 or IRF1 binding sites (Figure 5b). p65 co-transfection with the full-length NOS2 promoter and deletion 1 induced similar and significantly higher luciferase activities compared with controls. p65 co-transfection with deletion 2 also significantly increased the luciferase activity compared with controls, which may be attributable to the p65 binding site 3, but this activity was much lower than that of the full-length and deletion 1 constructs. This suggests that the deletion 1 region of −962 to +69 is essential for transcriptional activity of NOS2 mRNA (Figure 5b). p65 co-transfection with deletion 3 induced no luciferase activity, further supporting our bioinformatical prediction (Figure 5a,b). In contrast, IRF1 co-transfection with the different NOS2 promoter constructs did not increase luciferase activity (Figure 5b), confirming our observation that IRF1 does not participate in *G. parasuis* induction of NOS2 in 3D4/21 cells (Figure 4g). Subsequently, a set of mutations was introduced into the NOS2 promoter region containing the three p65 binding sites (binding site mutations 1, 2 and 3; Figure 5a) and their luciferase activities were compared with that of p65 co-transfection. Figure 5c shows that the NOS2 promoter with the wild-type p65 binding site (red triangle) exhibited significantly higher luciferase activity compared with the vector control. When these p65 binding sites were mutated separately (grey triangle), an extremely significant decrease of NOS2 promoter activity was observed (Figure 5c). An even lower level of NOS2 luciferase activity was detected when all three p65 binding sites were mutated concurrently (Figure 5c), suggesting that they function together to facilitate NOS2 transcription.

## 3. Discussion

NO is an important endogenous bioactive mediator of macrophages that mediates multiple effects during microbial infections [44]. Several pieces of evidence have shown that the production of NO in macrophages is stimulated by pathogens such as *S. aureus*, *Mycobacterium tuberculosis*, *Actinobacillus pleuropneumoniae* and *Blastomyces dermatitidis* [7,45,46,47]. Mammalian NO is derived from the catalyzation of L-arginine by NOS under complex oxidoreductase reactions [17]. A previous study indicated that NOS2 is transcribed at low abundance in porcine immune cells [48], but we demonstrated a high level of NO production in porcine alveolar macrophages in response to *G. parasuis* infection. *G. parasuis*-induced NOS2 expression mediates the generation of NO in porcine alveolar macrophages, which largely requires bacterial viability. We observed time-dependent and bacterial MOI-dependent NO production and NOS2 expression in 3D4/21 cells, which was dependent on bacterial viability. 

NOS2 activity can be regulated by cytokines or bacteria and affects GTP cyclohydrolase I, the key enzyme for BH4 synthesis, while BH4 is an essential component for NOS catalysis [12]. Similarly, IFN-γ is a positive stimulator for the generation of NO [49], and an IFN-γ-activated site was previously detected in the NOS2 promoter region in mouse macrophages [50]. In the present study, treatment with BH4 or IFN-γ during *G. parasuis* challenge greatly increased NOS2 transcription and significantly enhanced NO production. Noticeably, in the absence of *G. parasuis* challenge, these treatments had no effect on NO generation.

Our results provide solid evidence that *G. parasuis* activation of NF-κB signaling facilitates NOS2-mediated NO generation in 3D4/21 cells. Previous studies showed that IRF1 regulates the expression of murine NOS2 [51]. Here, we found that IRF1 was significantly upregulated in 3D4/21 cells upon *G. parasuis* infection. However, siRNA and dual-luciferase assays detected no regulatory effect of IRF1 on NOS2 expression; the luciferase assays also suggested that NOS2 transcriptional activity was independent of IRF1. Bioinformatics analysis identified four potential p65 binding sites in the promoter of NOS2, which is consistent with previous findings that NF-κB binds the human NOS2 promoter [52]. We also demonstrated the significant and time-dependent activation of NF-κB signaling in 3D4/21 cells in response to *G. parasuis* infection. Our pharmacological inhibitor assay subsequently revealed that NOS2 expression was largely increased by the activation of NF-κB signaling, and the luciferase assay showed that the p65 binding sites were all functionally involved in the transcriptional activity of the NOS2 promoter. These findings suggest the p65-triggered NF-κB signaling, but not IRF1-associated pathways, determines the NOS2-mediated generation of NO in porcine alveolar macrophages following *G. parasuis* challenge.

NOS2-mediated NO production contributes to proinflammatory activation in alveolar macrophages during *G. parasuis* infection. Importantly, NOS2-catalyzed reactive nitrogen intermediates have been reported to play important roles in mediating bactericidal activity, inhibiting bacterial infections and regulating inflammatory processes [53,54]. For example, uropathogenic *Escherichia coli* toxins induced the apoptosis of renal tubular epithelial cells via NOS, while the NO donor, SNP-mediated upregulation of heme oxygenase-1 prevented *E. coli*-induced apoptosis [39]. In enterohemorrhagic *E. coli* (EHEC), exogenously added or cellularly-derived NO inhibited spontaneous or mitomycin C-induced shiga-toxin (stx) mRNA transcription and Stx synthesis [55]. Additionally, the exogenous addition of NO restricted the intracellular growth of pathogenic *Rhodococcus equi* through depleting iron [32]. Moreover, studies have shown that NO inhibits EHEC adhesion of human intestinal epithelial cells [56], and suppresses *Staphylococcal* virulence by targeting the Agr quorum sensing system [57]. Here, we provide direct evidence that NO significantly inhibits the growth of *G. parasuis*, which could be recognized as an important protective strategy of macrophages. Meanwhile, NO exhibited potential pro-inflammatory activation effects by inducing the expression of multiple cytokines and chemokines in alveolar macrophages during *G. parasuis* infection. These findings, together, imply that alveolar macrophage-derived NO demonstrates important defensive effects against invading *G. parasuis*.

Bacterial pathogens have evolved a diverse range of strategies to overcome nitrative stresses and subvert host defenses. When exposed to a NO environment, *Histoplasma capsulatum* upregulates the expression of NO reductase to maintain or promote the secretion of virulence factors [58], while *S. enterica* Typhimurium induces a nitrative stress-resistance regulon that includes flavohemoglobin, a NO detoxifying enzyme required for bacterial virulence [59]. Although *S. aureus* lacks the NO-sensing transcriptional regulator NsrR, the bacterium uses its two-component system SrrAB to sense and respond to external nitrative stresses [41]. Using the iTRAQ approach, 13 proteins of *S. pneumoniae* were shown to be differentially expressed following low-concentration NO treatment, with 85% participating in metabolism or gene transcription [25]. We also investigated corresponding gene expression alterations in *G. parasuis* following low-concentration NO stimulation, and found that nitrate reductase genes (including *napA*, *napD*, *nrfC* and *nrfD*) were significantly upregulated. In contrast, cytochrome biosynthesis and assembly genes (*cydA* and *cydB*) were unaffected, and anaerobic metabolism genes, such as *pflA*, *pflB*, *adhE* and *nrdD* (excluding *nrdG*), were also unchanged in response to the NO challenge. These findings suggest that the nitrate reductase genes are probably utilized by *G. parasuis* to survive NO-mediated bacteriostatic effects. This may be an environmental stress adaptive response mechanism evolved by *G. parasuis* during its interaction with its host.

**Figure 6:**
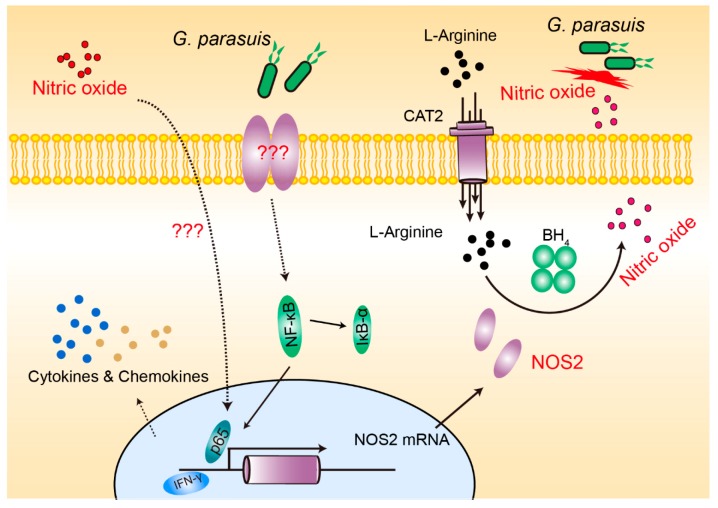
Schematic diagram of the pathways and molecules involved in this NO process in response to *G. parasuis* infection.

In summary, we herein demonstrated the significant induction of NO generation in porcine alveolar macrophages by *G. parasuis* infection. The bacterial activation of NF-κB signaling mediated NOS2 upregulation and largely contributed to NO production, which plays an important role in magnifying the inflammatory responses. While NO (or nitrite) exhibits detrimental effects on the growth of *G. parasuis*, the bacterium, correspondingly, selectively upregulates multiple nitrate reductase genes to overcome the environmental stress. Overall, our current work provides novel insights regarding alveolar macrophage-derived NO into the pathogenesis of *G. parasuis* and the stress-responsive strategy of the bacterium (Figure 6). These findings increase our understanding of *G. parasuis* pathogenesis from the perspective of interactions between pathogens and hosts, which will contribute to better prevention and control of this disease.

## 4. Materials and Methods

### 4.1. Bacterial Strains and Cell Culture

*G. parasuis* SH0165, whose whole genome we sequenced in a prior study [37], is a highly virulent serovar 5 strain clinically isolated from the lung of a diseased piglet in North China. Bacteria were cultured in tryptic soy broth (TSB) (Difco Laboratories, Detroit, MI, USA) or on tryptic soy agar (TSA) (Difco Laboratories) supplemented with 10 μg/mL nicotinamide adenine dinucleotide and 5% (v/v) inactivated newborn bovine serum (TSA/V/S or TSB/V/S, respectively) at 37 °C with 5% CO_2_. Cultured bacteria were washed and resuspended in RPMI 1640 medium (Life technologies, Grand Island, NY, USA) before experimental use. To prepare HK-SH0165, cultured bacteria were incubated at 70 °C for 20 min then washed and resuspended in RPMI 1640 medium. Complete killing of the bacteria was confirmed by plating on TSA/V/S plates.

The porcine alveolar macrophage cell line 3D4/21 was obtained from American Type Culture Collection (ATCC, Manassas, USA) (no. CRL-2843). Cells were cultured and maintained in RPMI 1640 medium supplemented with 2 mM L-glutamine, 1.5 g/L sodium bicarbonate, 4.5 g/L glucose, 10 mM HEPES, 1.0 mM sodium pyruvate, 0.1 mM nonessential amino acids and 10% fetal bovine serum (FBS) at 37 °C with 5% CO_2_. HEK293T cells (ATCC, no. CRL-11268) cell were cultured and maintained in Dulbecco’s modification of Eagle’s medium (DMEM) (Life technologies, Grand Island, NY, USA) supplemented with 10% FBS.

### 4.2. Porcine Alveolar Macrophage Infection

3D4/21 cells were seeded in 24-well plates at 2 × 10^5^ cells/well or in 60 mm dishes at 2 × 10^6^ cells/dish, measured by using red blood cell counting plate. Confluent cells were treated with or without the NOS2 specific inhibitor S-Methylisothiourea hemisulfate salt (S-MET, Sigma-Aldrich, St Louis, MO, USA), the NF-κB inhibitor BAY11-7082 (Sigma-Aldrich), the inhibitor of NF-κB kinase (IKK) β inhibitor CAY10657 (Cayman Chemical, Ann Arbor, MI, USA), IFN-γ (Kingfisher Biotech, St Paul, MN, USA) or tetrahydrobiopterin (BH4, Sigma-Aldrich) prior to the SH0165 challenge at a MOI of 100, 10 and 1, or for the HK-SH0165 challenge at a MOI of 100.

### 4.3. Growth Analysis of G. parasuis in Response to NO

An overnight culture of *G. parasuis* SH0165 was diluted to an optical density at 600 nm (OD_600_) of 1.0, as previously described [60]. The diluted bacterial suspension was inoculated 1:100 into fresh TSB/V/S medium with or without the NO donor sodium nitroprusside (SNP, Sigma-Aldrich) and incubated at 37 °C with circular agitation at 170 rpm/min for 24 h. The OD_600_ of the cultures was measured by using an Eppendorf Biospectrometer (Eppendorf, Hamburg, Germany) at 2 h intervals. The assay was performed independently in triplicate.

### 4.4. Nitrite Measurement and NO Detection

3D4/21 cells were infected with SH0165 or HK-SH0165; then, culture supernatants were collected to measure the nitrite content using Griess reagent according to the manufacturer’s instructions (Beyotime, Shanghai, China). Briefly, 50 μL of both Griess reagent I and reagent II were added to 50 μL of culture supernatants in 96 well-plates and incubated for 5 min at room temperature. The A_540_ was then measured with a microplate reader (Eppendorf, Hamburg, Germany). The nitrite content in each sample was calculated and normalized based on the standard curve of sodium nitrite with known concentrations.

Cellular NO production was detected using a cell-permeable fluorescent probe, (6’-Acetyloxy-5, 6-diamino-3-oxospiro [2-benzofuran-1, 9-xanthene]-3’-yl) acetate (DAF-2 DA, Abcam, Cambridge, MA, USA), as previously described [32]. In some assays, S-MET (50 μM), IFN-γ (50 ng/mL) or BH4 (20 μM) was maintained during the experiment. Cells were stained with 5 μM of DAF-2 DA for 30 min and washed with phosphate-buffered saline (PBS) to remove extracellular DAF-2 DA. Subsequently, cells were fixed with 4% paraformaldehyde for 30 min, washed three times with PBS and then stained with 4’,6-diamidino-2-phenylindole-dihychloride for 4 min. Cells in each well were finally washed with PBS, and then subjected to fluorescence intensity determination with a fluorescence microplate reader and image captured using a fluorescence microscope (EVOS FL Auto, Life Technologies corporation, Gaithersburg, MD, USA).

### 4.5. Plasmid Construction, Cell Transfection, Small Interfering RNA Transfection and the Dual Luciferase Reporter Assay

The coding sequence (CDS) of p65 (Gene ID: 100135665) and the interferon regulatory factor 1 gene (IRF1, Gene ID: 396611) were amplified from cDNAs of 3D4/21 macrophages using Prime STAR Max DNA polymerase (Takara Bio Inc., Shiga, Japan), and then cloned into the pcDNA3.1(+) vector (Invitrogen, Carlsbad, CA, USA), using *Kpn*I/*Eco*RI and *Xba*I restriction sites. The promoter region of NOS2 (Gene ID: 396859) was amplified and cloned into the pGL3-luciferase plasmid (Promega, Madison, WI, USA), using *Nhe*I and *Hind*III restriction sites. Plasmid cloning with p65 or IRF1 CDS and plasmids with the NOS2 promoter region were co-transfected into HEK293T cells using the jetPRIME transfection regent (Polyplus transfection, Illkirch, France) for subsequent dual luciferase reporter analysis. Briefly, cells were seeded in 24-well plates at 1 × 10^5^ cells/well and incubated for 24 h. A total of 50 μL of jetPRIME buffer; 200 ng of plasmid DNA (pGL3-NOS2, pcDNA3.1(+)-p65, or pcDNA3.1(+)-IRF1); and 20 ng of control plasmid DNA pRL-TK, were mixed; then, 2 μL of jetPRIME reagent was added into the mixture and incubated at room temperature for 10 min prior to adding into 24 well-plates. Cells were cultured for 24 h and luciferase activity was measured using the dual-luciferase reporter assay system (Promega, Madison, WI, USA) according to the manufacturer’s recommendations. Relative luciferase activity was calculated by the ratio of reporter and control activity (Firefly fluorescence) to that of control activity (Renilla fluorescence).

In the IRF1 small interfering (si) RNA knockdown assay, 40 pmol of IRF1 siRNA oligonucleotide (GenePharma, Suzhou, China) was transfected into 3D4/21 cells using jetPRIME regents. IRF1 and negative control siRNA sequences are shown in Table 1.

### 4.6. Western Blotting

Cells were seeded in 60-mm dishes at 2 × 10^6^ cells/dish and grown until confluent. After specific treatment, cells were collected and lysed in 500 μL of Western blot lysis buffer (Beyotime, Shanghai, China) supplemented with the proteinase inhibitor phenylmethylsulfonyl fluoride (Sigma-Aldrich). The protein concentration was determined by the bicinchoninic acid assay (BIOSHARP, Hefei, China), and equivalent amounts of protein were separated on sodium dodecyl sulfate polyacrylamide gel electrophoresis (5%–12% polyacrylamide) and then transferred to a polyyinylidene difluoride membrane. The membrane was blocked with 5% bovine serum albumin (BSA) in 0.1 M Tris-buffer saline (pH 7.5) containing 0.05% Tween-20 (TBST) for at least 1 h, and then probed overnight with appropriate primary antibodies against as follows: IκB-α, p-p65, p65 or β-actin (1:2000 in 5% BSA in TBST; Cell Signaling Technology, Danvers, MA, USA). They were then incubated with anti-mouse or anti-rabbit horseradish peroxidase-conjugated secondary antibodies for 2 h at 37 °C. Blots were finally visualized with ECL reagents (Bio-Rad, Hercules, CA, USA) via chemiluminescence. To compare reaction intensities, average band densities were determined using Quantity One software (Bio-Rad).

### 4.7. RNA Isolation and Real-Time PCR

Total RNA from cells or bacteria was extracted using Trizol reagent (Invitrogen, Carlsbad, CA, USA) according to the manufacturer’s guidelines. DNase I (New England Biolabs, Ipswich, MA, USA) was applied to remove residual genomic DNA for the following reverse transcription (RT) reactions (Takara Bio Inc., Shiga, Japan). mRNA transcription levels were determined using the Power SYBR Green PCR master mix (Applied Biosystems, Foster City, CA, USA) according to standard instructions. Primers used for RT-PCR and real-time PCR are listed in Table 1. GAPDH was used as the internal reference for 3D4/21 cells and the specific 16S rRNA was used as the control for *G. parasuis*. The 2^-ΔΔCT^ method was used to calculate relative gene expression.

### 4.8. Statistical Analysis

The significance of the differences between each group was analyzed by two-way analysis of variance (ANOVA) embedded in GraphPad Prism version 6.0 and Student’s *t*-tests. The *p*-value < 0.05 (*) was statistically significant, and both *p* < 0.01 (**) and *p* < 0.001 (***) were considered extremely significant.

## Figures and Tables

**Table 1:** Primers used in this study.

Primer Name	Primer Sequence (5’ to 3’) ^1^
NOS2-Promoter-F (*Nhe*I)	CATGCTAGCCAAGCATGCGTGCATATCTCC
NOS2-Promoter-R (*Hind*III)	CCCAAGCTTGAGGTGACCTGACTCACGGA
Promoter-Deletion 1-F (*Nhe*I)	CATGCTAGCAAAGAAATGGGTTGTGGGTG
Promoter-Deletion 2-F (*Nhe*I)	CATGCTAGCCACCCTGTCTTGGTCCTTTG
Promoter-Deletion 3-R (*Hind*III)	CCCAAGCTTAAGGGACAGAGGAGAGCATCTC
Promoter-Mutation 1-F	AAAAACCTTTTGGATCCGGAGAGATATGG
Promoter-Mutation 1-R	AAAGGTTTTTACTCTCGTCCAGGAATCTCCA
Promoter-Mutation 2-F	AAGAACCTTTAGGCTAGGGGTTGAATCGG
Promoter-Mutation 2-R	AAAGGTTCTTATATGCCACGGGAGCGGC
Promoter-Mutation 3-F	AAAGGTCTTACTGTGAAGTTTTTATCCATGGGT
Promoter-Mutation 3-R	TAAGACCTTTGTCATGGCGCAGTGGTTAAC
p65-F (*Kpn*I)	GGGGTACCATGGACGACCTCTTCCCCCT
p65-R (*Xba*I)	GCTCTAGATTAGGAGCTGATCTGACTCA
IRF1-F (*Eco*RI)	CCGGAATTCATGCCCATCACTCGGATG
IRF1-R (*Xba*I)	GCTCTAGACTACGGTGCACAAGGAATG
qIRF1-F	ACCGTGTGCCATCAGTAGTA
qIRF1-R	CCTCCTCGTCCTCATCTGTT
qGAPDH-F	CACAGTCAAGGCGGAGAAC
qGAPDH-R	CGTAGCACCAGCATCACC
qNOS1-F	GTCCAGACCTCAGAGACAACT
qNOS1-R	CCACGCAGAACACATCACA
qNOS2-F	AGCCTCTGGACCTCAACAA
qNOS2-R	GCTGGATTGCGGACTCTG
qNOS3-F	GATGCCGAAGCGAGTGAAG
qNOS3-R	CACCAACACCAGCGTCTC
qCAT1-F	TGAAGGCTCTCGTGGACAT
qCAT1-R	GGACAGTATTTGGGTTGCTCATAA
qCAT2-F	AGCCTGAGAGCAAGACCAA
qCAT2-R	CGTAGCCGAAGTAGATGAAGAAG
qIL-6-F	TTCAGTCCAGTCGCCTTCT
qIL-6-R	TCACACATCTCCTTTCTCATTGC
qGM-CSF-F	AGACTCGCCTGAACCTGTA
qGM-CSF-R	TGCTGCTCATAGTGCTTGG
qCCL-2-F	GTCACCTGCTGCTATACACTTAC
qCCL-2-R	ATCACTGCTTCTTTAGGACACTTG
cydA-F	AGGCTTAATGGCGTTCTTCTTAG
cydA-R	GCATCCAACCGTTAGCAACTA
cydB-F	CAATGTTGAACGCCGTGTAATG
cydB-R	GGAAGAATAATGCCGCTAGAACT
pflA-F	GCCACAACCGTGACACTT
pflA-R	CGCCACCTGATGCTGTTAC
pflB-F	GCGATTGCCTGCTGTGTAA
pflB-R	GCCATTGATTGCGTATAGTAAGGT
adhE-F	CAACAAGGCTGGCGAGTAAT
adhE-R	GGCTGAGTTAGTCACATCAAGTT
nrdD-F	CGGAGGTTCGGCATTACATC
nrdD-R	TGGTCACAGGTGTTACATAAGGT
nrdG-F	CTTGGAAGCGGTCAGATTCTC
nrdG-R	AATAACAATTCAGGCAACCACTCT
napA-F	CGCTTAACCTCGCCAATGT
napA-R	TGATTGACCTGAAGTGAACATACC
napD-F	GTGAAGCAAGCCTTAACTGAGT
napD-R	CAGCGACACCACGATAACC
nrfC-F	CTGTGTTGGTTGTGCTTATTGC
nrfC-R	CGAATGTTAGTGCTTTGGTTGGA
nrfD-F	GGATTCAACCATTGCGATCTATCT
nrfD-R	AGCGGTGACATTATTGCCATT
16S rRNA-F	TGAAGTCGGAATCGCTAGTA
16S rRNA-R	CCTACGGTTACCTTGTTACG
siIRF1-1-F	CCAACUUUCGCUGUGCCAUTT
siIRF1-1-R	AUGGCACAGCGAAAGUUGGTT
siIRF1-2-F	GGACAUUGAACAGGCCCUUTT
siIRF1-2-R	AAGGGCCUGUUCAAUGUCCTT

^1^ All restriction sites are indicated in Red.
